# Heart Rate Variability in Coexisting Diabetes and Hypertension

**DOI:** 10.5935/abc.20180118

**Published:** 2018-07

**Authors:** Paula F. Martinez, Marina P. Okoshi

**Affiliations:** 1 Faculdade de Fisioterapia, Universidade Federal de Mato Grosso do Sul, Campo Grande - Brazil; 2 Departamento de Clínica Médica - Faculdade de Medicina de Botucatu, Universidade Estadual de São Paulo (UNESP), Botucatu, SP – Brazil

**Keywords:** Hypertension, Diabetes Mellitus, Chronic Disease, Heart Rate, Autonomic Nervous System

The autonomic nervous system regulates heart rate through sympathetic and parasympathetic response to different stimuli. The resultant fluctuation between intervals of consecutive heart beats, called heart rate variability (HRV), is a valuable tool to assess autonomic nervous system activity.^1^ A decrease in HRV is a marker of reduced parasympathetic and increased sympathetic tone and has long been considered to negatively impact the prognosis in cardiovascular disease.^2^

In 1996, the European Society of Cardiology and the North American Society of Pacing and Electrophysiology suggested standards for evaluation, physiological interpretation, and clinical use for time- and frequency-domain HRV analysis in short- and long-term recordings.^3^ Some nonlinear measures have been suggested to work better than traditional measures in predicting future adverse events in several patient groups. More recently, newer computational tools have been derived from nonlinear dynamics and complex systems.^4^ Although the physiological background of nonlinear measures of HRV is less understood than the conventional measures, it is speculated that nonlinear dynamics could provide better understanding on nonlinear behavior commonly occurring within human systems due to their complex dynamic nature.^5,6^ In accordance, a good agreement between some non-linear HRV measures and the Framingham cardiovascular risk score was observed, suggesting that they could be used for screening cardiovascular risk.^7^ In 2015, the e-Cardiology Working Group of the European Society of Cardiology and the European Heart Rhythm Association launched a critical review of new methodologies for analyzing HRV, including entropy rate, fractal scaling and *Poincaré* plot, and their application in different physiological and clinical studies.^8^

Alterations in HRV time and frequency domain indices have been frequently observed in chronic diseases, such as diabetes and hypertension, and associated with cardiac autonomic dysfunction.^9,10^ Since co-existence of diabetes mellitus and systemic arterial hypertension is very common, some studies have compared HRV between type 2 diabetic patients with and without hypertension, and found contradictory results using time-and frequency-domain HRV analysis.^11-13^ However, non-linear dynamics for HRV analysis in type 2 diabetes and hypertension co-existence is still to be explored.

In this issue of the *Arquivos Brasileiros de Cardiologia*, Bassi et al.^14^ published a study evaluating the influence of systemic arterial hypertension on cardiac autonomic modulation and cardiopulmonary capacity in type 2 diabetic patients. Diabetes subjects were assigned to a normotensive (n = 32, age = 51 ± 7.5 years) or a hypertensive group (n = 28, age = 51 ± 6.9 years). Both groups had a poor glycemic control (normotensive group: glycated hemoglobin = 8.00 ± 2.14%; hypertensive group: glycated hemoglobin = 8.70 ± 1.60%; p = 0.39) and the hypertensive group had a higher insulin resistance (normotensive group: insulin resistance index (HOMA-IR) = diabetes 4.0 ± 4.0; hypertensive group: HOMA-IR = 8.0 ± 6.6; p = 0.02). The authors found that hypertensive and diabetic subjects had lower SD1 (derived from *Poincaré* plot) and Shannon entropy, both non-linear measures of HRV, in comparison to non-hypertensive diabetic patients. In addition, SD2 (derived from *Poincaré* plot) and approximate entropy correlated negatively with exercise capacity variables.

Although a healthy control group was not evaluated, the results suggest that systemic arterial hypertension further impairs HRV in diabetic patients. These data reinforce epidemiological findings showing that the combination of diabetes mellitus and hypertension induces greater cardiac remodeling than either condition alone.^15^ Furthermore, heart failure is more prevalent in patients with both diseases. Additional studies are needed to establish the role of autonomic nerve dysfunction as a predictor of poor prognosis in patients with co-existing diabetes and hypertension.

## References

[1] Xhyheri B, Manfrini O, Mazzolini M, Pizzi C, Bugiardini R (2012). Heart rate variability today. Prog Cardiovasc Dis.

[2] Huikuri HV, Stein PK (2013). Heart rate variability in risk stratification of cardiac
patients. Prog Cardiovasc Dis.

[3] Heart rate variability: standards of measurement, physiological
interpretation and clinical use (1996). Task Force of the European Society of Cardiology and the North
American Society of Pacing and Electrophysiology. Eur Heart J.

[4] Mirvis DM, Goldberger AL, Bonow RO, Mann DL, Zipes DP, Libby P, Braunwald E (2012). Electrocardiography. Braunwald's heart disease: a textbook of cardiovascular
medicine.

[5] Perkiomaki JS (2011). Heart rate variability and non-linear dynamics in risk
stratification. Front Physiol.

[6] Higgins JP (2002). Nonlinear systems in medicine. Yale J Biol Med.

[7] Jelinek HF, Md Imam H, Al-Aubaidy H, Khandoker AH (2013). Association of cardiovascular risk using non-linear heart rate
variability measures with the Framingham risk score in a rural
population. Front Physiology.

[8] Sassi R, Cerutti S, Lombardi F, Malik M, Huikuri HV, Peng CK (2015). Advances in heart rate variability signal analysis: joint
position statement by the e-Cardiology ESC Working Group and the European
Heart Rhythm Association co-endorsed by the Asia Pacific Heart Rhythm
Society. Europace.

[9] Wilson LC, Peebles KC, Hoye NA, Manning P, Sheat C, Williams MJ (2017). Resting heart rate variability and exercise capacity in type 1
diabetes. Physiol Rep.

[10] Anaruma CP, Ferreira MJ, Sponton CH, Delbin MA, Zanesco A (2016). Heart rate variability and plasma biomarkers in patients with
type 1 diabetes mellitus: effect of a bout of aerobic
exercise. Diabetes Res Clin Pract.

[11] Takahashi N, Nakagawa M, Saikawa T, Ooie T, Yufu K, Shigematsu S (2001). Effect of essential hypertension on cardiac autonomic function in
type 2 diabetic patients. J Am Coll Cardiol.

[12] Istenes I, Korei AE, Putz Z, Németh N, Martos T, Keresztes K (2014). Heart rate variability is severely impaired among type 2 diabetic
patients with hypertension. Diabetes Metab Res Rev.

[13] Solanki JD, Basida SD, Mehta HB, Panjwani SJ, Gadhavi BP (2017). Comparative study of cardiac autonomic status by heart rate
variability between under-treatment normotensive and hypertensive known type
2 diabetics. Indian Heart J.

[14] Bassi D, Cabiddu R, Mendes RG, Tossini N, Arakelian VM, Caruso FC (2018). Efeitos da coexistência de diabetes tipo 2 e
hipertensão sobre a variabilidade da frequência
cardíaca e capacidade cardiorrespiratória. Arq Bras Cardiol.

[15] Rosa CM, Xavier NP, Campos DH, Fernandes AA, Cezar MD, Martinez PF (2013). Diabetes mellitus activated fetal gene program and intensifies
cardiac remodeling and oxidative stress in aged spontaneously hypertensive
rats. Cardiovasc Diabetol.

